# Linking Global Antioxidant Assays with Targeted HPLC Profiling of Prenylated Flavonoids in *Humulus lupulus* L. Extracts Obtained by Accelerated Solvent Extraction

**DOI:** 10.3390/molecules31030562

**Published:** 2026-02-05

**Authors:** Nora Haring, Blažena Drábová, Milan Chňapek

**Affiliations:** Faculty of Biotechnology and Food Sciences, Slovak University of Agriculture in Nitra, Tr. A. Hlinku 2, 949 76 Nitra, Slovakia; qharing@uniag.sk (N.H.); blazena.drabova@uniag.sk (B.D.)

**Keywords:** accelerated solvent extraction (ASE), extraction temperature, HPLC-DAD, *Humulus lupulus* L., prenylated flavonoids, total antioxidant capacity (TAC), total phenolic content (TPC)

## Abstract

Accelerated solvent extraction (ASE) is widely used for recovering bioactive compounds from hops; however, the extent to which global antioxidant assays reflect changes in molecular composition remains unclear. This study evaluated the relationship between global antioxidant parameters and targeted profiling of prenylated flavonoids in hop extracts obtained under different ASE conditions. Total antioxidant capacity (TAC), total phenolic content (TPC), and concentrations of xanthohumol, isoxanthohumol, and 8-prenylnaringenin were determined in extracts prepared using different solvents, extraction temperatures, and homogenization approaches. Global antioxidant parameters responded consistently to technological factors and exhibited a strong mutual correlation. In contrast, their correlations with individual prenylated flavonoids were moderate, indicating that global assays capture only part of the variability associated with specific bioactive compounds. Extraction temperature emerged as a key modulating factor, inducing compound-specific and partly non-linear responses that were not fully reflected by global antioxidant methods. Principal component analysis confirmed a shared chemical trend linking global and targeted parameters while separating extraction temperature as an independent technological driver. Overall, global antioxidant assays provide a robust but simplified assessment of hop extract quality. Their combination with targeted chromatographic analysis enables more accurate interpretation of extraction behavior and supports informed process optimization aimed at preserving and recovering bioactive compounds.

## 1. Introduction

Hops (*Humulus lupulus* L.) have traditionally been used in brewing as a source of bitter and aromatic compounds; however, increasing attention has been paid in recent decades to their content of biologically active substances with potential health benefits. Among the most important of these are polyphenols and prenylated flavonoids, which exhibit antioxidant, anti-inflammatory, antimicrobial, and hormonal activities [1,2,3]. The most extensively studied compounds include xanthohumol, isoxanthohumol, and 8-prenylnaringenin, which are localized in the lupulin glands of hop cones and significantly contribute to the biological potential of hop extracts [1,4].

The recovery of these bioactive compounds from hops is strongly influenced by extraction conditions, particularly the type of solvent, extraction temperature, and the method of plant material pretreatment. Numerous studies have demonstrated that elevated temperature and pressure can markedly enhance extraction efficiency; however, they may also promote chemical transformations or degradation of thermolabile compounds [2,5,6]. For this reason, accelerated solvent extraction (ASE) is considered a promising tool for the systematic investigation of bioactive compound behavior under controlled high-temperature and high-pressure conditions [5,7].

To evaluate the quality of hop extracts, global analytical methods such as the determination of total antioxidant capacity (TAC) and total phenolic content (TPC) are frequently employed. These approaches provide rapid information on the antioxidant potential of extracts; however, their lack of specificity represents a significant limitation, as they do not directly reflect the concentrations of individual bioactive molecules [8,9]. In the case of hops, this limitation is particularly relevant, since prenylated flavonoids exhibit chemical behavior and biological activity distinct from those of other phenolic constituents [1,2].

While accelerated solvent extraction has been widely applied for the isolation of phenolic compounds from plant matrices, its use alone does not resolve how global antioxidant responses relate to compound-specific behavior under varying extraction conditions. In chemically complex systems such as hop extracts, global antioxidant assays integrate the cumulative contribution of multiple phenolic and non-phenolic constituents, which may respond differently to changes in extraction parameters. As a result, strong antioxidant responses at the extract level do not necessarily imply proportional preservation or enrichment of specific bioactive compounds, particularly thermally sensitive prenylated flavonoids.

Despite the growing number of studies focused on the extraction of polyphenols and prenylated flavonoids from hops, a comprehensive evaluation that systematically links global antioxidant assays with targeted chromatographic analysis of individual prenylated flavonoids under varying extraction conditions is still lacking. In particular, when using high-pressure extraction techniques such as ASE, it remains insufficiently explored to what extent global parameters such as TAC and TPC reflect actual changes in the molecular composition of the extracts.

The aim of this study was therefore to systematically investigate the effects of solvent type, extraction temperature, and homogenization method on the antioxidant potential and chemical composition of hop extracts. By combining global analytical approaches (TAC, TPC) with targeted HPLC-DAD analysis of prenylated flavonoids (xanthohumol, isoxanthohumol, and 8-prenylnaringenin), we assessed the extent to which these methods provide a consistent representation of extract quality.

From a practical perspective, the study seeks to clarify under which extraction scenarios global antioxidant assays provide reliable guidance for process optimization and quality screening, and when targeted chromatographic analysis becomes essential for meaningful process control and product standardization. The results contribute to a deeper understanding of the interpretative limits of global antioxidant assays and provide a methodological framework for optimizing extraction strategies aimed at the recovery of hop-derived bioactive compounds.

## 2. Results

This section summarizes the experimental data obtained from hop extracts prepared under different accelerated solvent extraction conditions. Global antioxidant parameters were evaluated first and subsequently complemented by targeted chromatographic, correlation, and multivariate analyses.

### 2.1. Effect of Extraction Parameters on Global Antioxidant Indicators (TAC and TPC)

This section summarizes the behavior of global antioxidant indicators in hop extracts obtained under different accelerated solvent extraction (ASE) conditions. Total antioxidant capacity (TAC) and total phenolic content (TPC) were evaluated as integrative parameters reflecting the combined contribution of multiple antioxidant-active constituents.

#### 2.1.1. General Trends in TAC and TPC

Both TAC and TPC were significantly affected by extraction temperature, homogenization method, solvent type, hop variety, and processing technology. Across all hop varieties and extraction conditions, the two global indicators exhibited highly consistent responses to technological parameters, providing a robust basis for subsequent comparison with targeted chromatographic data.

TAC and TPC values generally increased with increasing extraction temperature from 50 °C to 100–150 °C, followed by stabilization or a slight decrease at 200 °C. This trend was consistently observed for both extraction solvents and all hop varieties, confirming extraction temperature as the dominant factor governing the global antioxidant response under ASE conditions.

#### 2.1.2. Effect of Extraction Temperature

Multifactor analysis of variance identified extraction temperature as the primary factor influencing both TAC and TPC. For TAC, extraction temperature accounted for approximately one third of the total variance (η^2^ ≈ 0.34; *p* < 0.001), with a similarly strong effect observed for TPC (η^2^ ≈ 0.36; *p* < 0.001). Maximum values of both parameters were consistently reached within the temperature range of 100–150 °C across all hop varieties and extraction solvents.

At 50 °C, extraction efficiency was limited, whereas extraction at 200 °C resulted in stabilization or a slight decrease in TAC and TPC values, indicating that further temperature increase did not proportionally enhance the global antioxidant response.

#### 2.1.3. Effect of Homogenization Method and Solvent Type

The homogenization method represented the second most influential technological factor after extraction temperature. Cryogenic pulverization resulted in higher TAC and TPC values compared to mechanical homogenization across most hop varieties, with increases typically ranging between 10% and 20%. The magnitude of this effect varied depending on hop variety, reflecting differences in matrix accessibility.

Solvent type exerted a statistically significant but comparatively minor effect on global antioxidant indicators. Ethanolic extracts generally exhibited slightly higher TAC and TPC values than methanolic extracts; however, the contribution of solvent type to overall variability was substantially lower than that of extraction temperature and homogenization method. At elevated temperatures, differences between ethanol and methanol became less pronounced.

#### 2.1.4. Effect of Cryogenic Lupulin Separation (CLS)

Hop material obtained by cryogenic lupulin separation (CLS) consistently exhibited lower TAC and TPC values compared to conventionally pelletized hop material. This reduction was strongly variety-dependent and most pronounced for Centennial CLS samples, irrespective of extraction solvent or homogenization method. Although cryogenic pulverization partially mitigated this effect, TAC and TPC values of CLS material did not reach those observed for conventional pellets.

#### 2.1.5. Summary of Global Analytical Method Behavior

Overall, global antioxidant indicators responded systematically and reproducibly to technological parameters, with extraction temperature exerting the strongest influence, followed by homogenization method and solvent type. While TAC and TPC provided coherent trends across all extraction conditions, these integrative parameters do not resolve the contribution, stability, or transformation of individual bioactive compounds.

Complete datasets for TAC and TPC obtained under all extraction conditions and for all hop varieties are provided in the Supplementary Materials (Tables S1–S4). The implications of these global trends in relation to compound-specific behavior are addressed in subsequent sections.

### 2.2. Quantitative Profiling of Prenylated Flavonoids Determined by HPLC-DAD

Quantitative HPLC-DAD analysis was used to assess the behavior of individual prenylated flavonoids—xanthohumol (XN), isoxanthohumol (IXN), and 8-prenylnaringenin (8-PN)—in hop extracts obtained under different accelerated solvent extraction (ASE) conditions. The concentrations of all three analytes were significantly affected by extraction temperature, solvent type, homogenization method, and hop variety.

To maintain clarity and readability of the main text, representative quantitative results for the Polaris variety are presented below. Complete datasets covering all hop varieties, extraction conditions, and processing variants are provided in the Supplementary Materials (Tables S5–S7), including the full range of observed concentrations and inter-varietal variability.

#### 2.2.1. General Behavior of Prenylated Flavonoids During Extraction

All three flavonoids exhibited compound-specific responses to extraction parameters. Across all conditions, ethanolic extracts generally yielded higher concentrations of prenylated flavonoids than methanolic extracts, particularly at elevated extraction temperatures and following cryogenic homogenization.

Extraction temperature exerted a pronounced influence on flavonoid profiles, with distinct and non-uniform trends observed among individual compounds. While intermediate temperatures favored the recovery of xanthohumol, higher temperatures promoted the formation of isoxanthohumol. In contrast, 8-prenylnaringenin was detected at substantially lower concentrations and exhibited a narrower temperature optimum.

#### 2.2.2. Xanthohumol (XN)

Xanthohumol concentrations were highest in ethanolic extracts of cryogenically homogenized Polaris samples, with maximum values observed at an extraction temperature of 150 °C. At lower temperatures (50–100 °C), XN concentrations were slightly reduced, whereas a pronounced decrease in concentration was observed at 200 °C.

Methanolic extracts consistently yielded lower XN concentrations compared to ethanolic extracts across all temperatures. Cryogenic homogenization enhanced XN recovery relative to mechanical homogenization under all evaluated extraction conditions. Multifactor analysis of variance confirmed significant effects of hop variety, solvent type, extraction temperature, and homogenization method on xanthohumol yield.

#### 2.2.3. Isoxanthohumol (IXN)

Isoxanthohumol exhibited a strong temperature dependence, with concentrations increasing progressively across the investigated temperature range and reaching maximum values at 200 °C. This trend was observed for both extraction solvents, with ethanolic extracts consistently yielding higher IXN concentrations than methanolic extracts.

Cryogenic homogenization resulted in a moderate increase in IXN yield compared to mechanical homogenization; however, the effect of extraction temperature was more pronounced than that of sample pretreatment. Statistical evaluation confirmed significant effects of all main factors and their interactions on isoxanthohumol concentrations.

#### 2.2.4. 8-Prenylnaringenin (8-PN)

Compared with XN and IXN, 8-prenylnaringenin occurred at substantially lower concentrations in all hop extracts. The highest 8-PN levels were detected at an extraction temperature of 100 °C in ethanolic extracts following cryogenic homogenization. At higher temperatures (150–200 °C), a gradual decrease in 8-PN concentration was observed.

Methanolic extracts consistently exhibited lower 8-PN concentrations than ethanolic extracts. Cryogenic homogenization had a positive but less pronounced effect on 8-PN yield compared to its impact on xanthohumol recovery.

#### 2.2.5. Summary of the Effects of Technological Factors on Prenylated Flavonoids

Multifactor statistical analysis confirmed that the yields of all three prenylated flavonoids were significantly influenced by hop variety, solvent type, extraction temperature, and homogenization method, as well as by their interactions. Ethanol and cryogenic homogenization consistently promoted higher concentrations of all analyzed prenylated flavonoids.

Extraction temperature emerged as the dominant technological factor shaping compound-specific profiles, with individual prenylated flavonoids exhibiting distinct and partially opposing temperature-dependent trends. These quantitative results provide the basis for subsequent integration with global antioxidant parameters and correlation-based interpretation.

### 2.3. Correlation Analysis Between Global Antioxidant Indicators and Prenylated Flavonoids

Correlation analysis was performed to evaluate the relationships between global antioxidant indicators (TPC and TAC) and the concentrations of individual prenylated flavonoids determined by HPLC-DAD. Pearson correlation coefficients were used to assess linear associations, while Spearman rank correlations were applied as a complementary non-parametric measure to verify trend consistency in the presence of non-linear temperature responses.

#### 2.3.1. Relationship Between TAC and TPC

A strong positive correlation was observed between TPC and TAC across all hop varieties and extraction conditions, confirming the coherent response of both global assays to changes in extraction parameters. Pearson correlation coefficients consistently exceeded *r* = 0.85 (*p* < 0.001), indicating that both indicators captured closely related aspects of the overall antioxidant potential of hop extracts.

This strong association supports the use of TPC and TAC as complementary global indicators for comparative assessment of extraction efficiency under ASE conditions.

#### 2.3.2. Correlation Between Global Antioxidant Indicators and Xanthohumol

**Figure 1:**
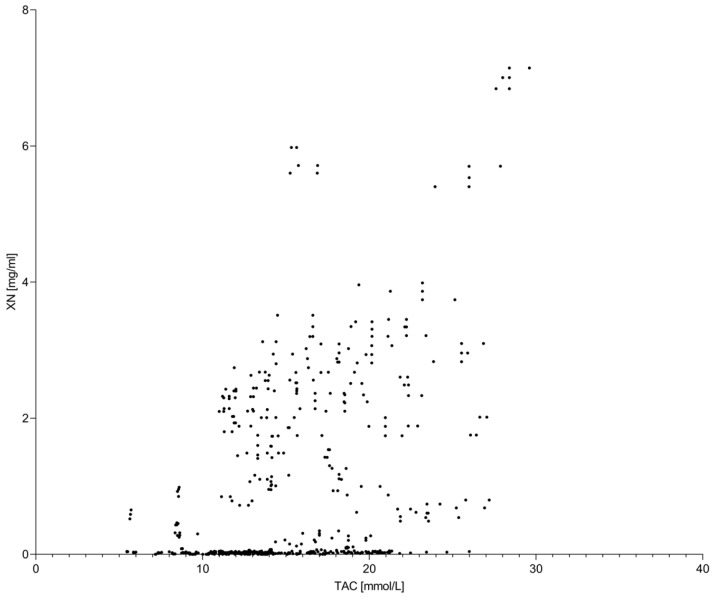
Relationship between total antioxidant capacity (TAC) and xanthohumol (XN) concentration in hop extracts. Each data point represents an individual extract obtained under different accelerated solvent extraction conditions, including variations in extraction temperature, solvent type, and homogenization method.

**Figure 2:**
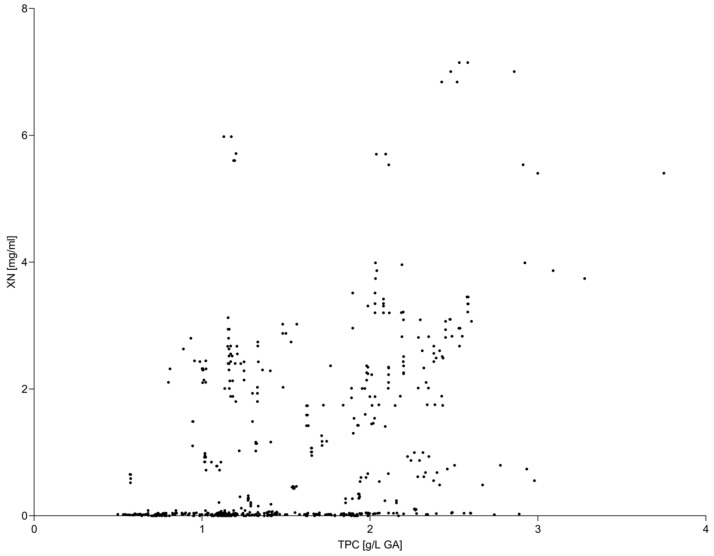
Relationship between total phenolic content (TPC) and xanthohumol (XN) concentration in hop extracts. Each data point represents an individual extract obtained under different accelerated solvent extraction conditions, including variations in extraction temperature, solvent type, and homogenization method. The scatter plot illustrates a moderate association between TPC and XN, indicating that changes in total phenolic content are not directly proportional to xanthohumol concentration across the investigated extraction conditions.

Moderate correlations were observed between global antioxidant indicators and xanthohumol concentration (Figure 1 and Figure 2). Pearson correlation coefficients ranged from weak to moderate depending on hop variety and extraction conditions, with Spearman correlations showing comparable trends. These results indicate that increases in TPC and TAC were not directly proportional to changes in xanthohumol concentration across the investigated temperature range.

Notably, extraction temperatures associated with maximum TPC and TAC did not necessarily coincide with optimal preservation of xanthohumol, reflecting the compound-specific thermal sensitivity of this prenylated flavonoid.

#### 2.3.3. Correlation Between Global Antioxidant Indicators and Isoxanthohumol

**Figure 3:**
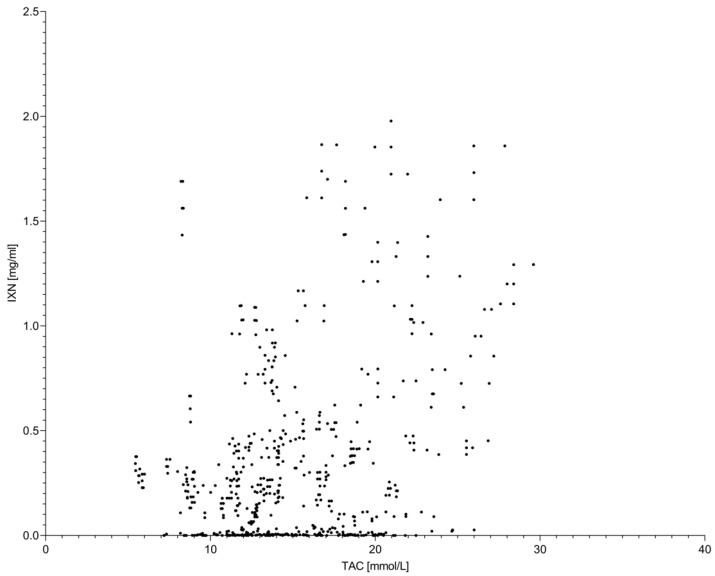
Relationship between total antioxidant capacity (TAC) and isoxanthohumol (IXN) concentration in hop extracts. Each data point represents an individual extract obtained under different accelerated solvent extraction conditions, including variations in extraction temperature, solvent type, and homogenization method. The scatter plot illustrates a moderate association between TAC and IXN, indicating that changes in global antioxidant capacity partially reflect variations in isoxanthohumol concentration across the investigated extraction conditions.

**Figure 4:**
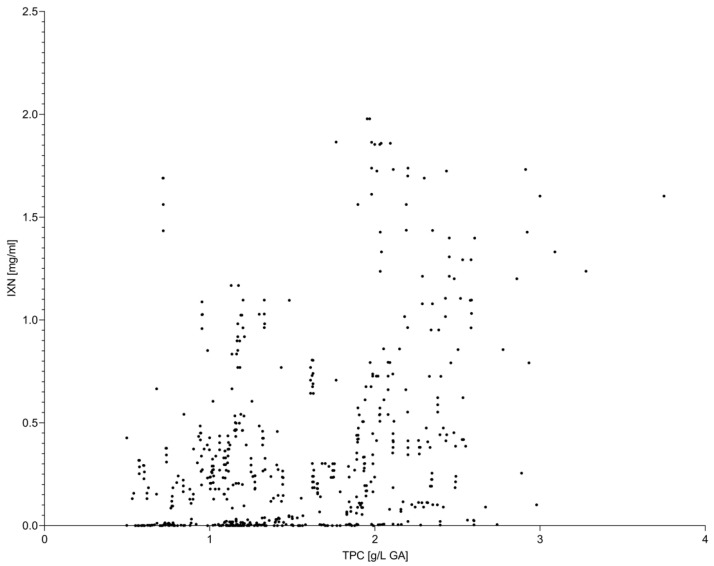
Relationship between total phenolic content (TPC) and isoxanthohumol (IXN) concentration in hop extracts. Each data point represents an individual extract obtained under different accelerated solvent extraction conditions, including variations in extraction temperature, solvent type, and homogenization method. The scatter plot demonstrates a moderate association between TPC and IXN, suggesting that total phenolic content captures only part of the variability associated with isoxanthohumol across the investigated extraction conditions.

Isoxanthohumol exhibited weak to moderate positive correlations with both TPC and TAC (Figure 3 and Figure 4). Correlation strength increased at higher extraction temperatures, consistent with the observed temperature-dependent increase in IXN concentration. However, overall correlation coefficients remained lower than those observed between TPC and TAC, indicating that IXN represents only a partial contributor to the global antioxidant response.

#### 2.3.4. Correlation Between Global Antioxidant Indicators and 8-Prenylnaringenin

**Figure 5:**
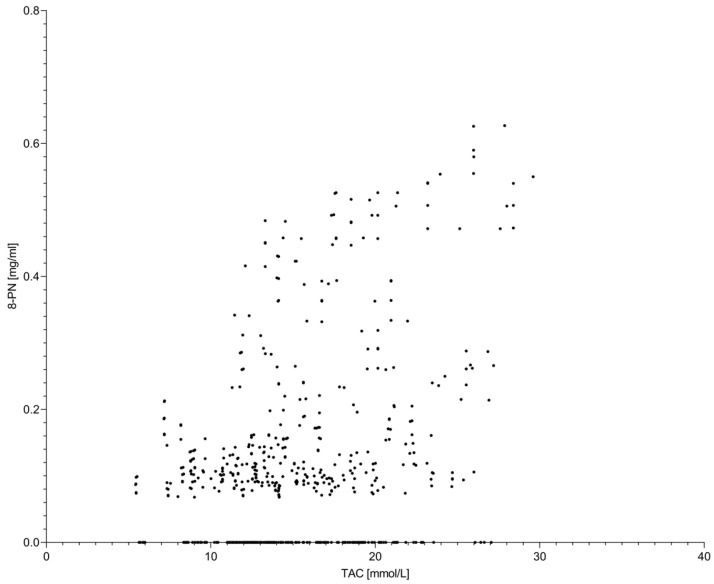
Relationship between total antioxidant capacity (TAC) and 8-prenylnaringenin (8-PN) concentration in hop extracts. Each data point represents an individual extract obtained under different accelerated solvent extraction conditions, including variations in extraction temperature, solvent type, and homogenization method. The scatter plot indicates a weak association between TAC and 8-PN, reflecting the low abundance and limited contribution of this prenylated flavonoid to the overall antioxidant capacity of hop extracts.

Correlations between global antioxidant indicators and 8-prenylnaringenin were generally weak (Figure 5). Due to the low absolute concentrations of 8-PN and its narrow temperature optimum, changes in its concentration exerted only a minor influence on TPC and TAC values. Pearson and Spearman coefficients were consistent in indicating limited association between 8-PN and global antioxidant parameters.

#### 2.3.5. Summary of Correlation Analysis

Overall, correlation analysis demonstrated that while TPC and TAC are strongly interrelated, their associations with individual prenylated flavonoids are moderate to weak and highly dependent on extraction conditions. These findings indicate that global antioxidant assays integrate the combined contribution of multiple extract constituents and do not selectively reflect changes in specific prenylated flavonoids.

Complete correlation matrices and statistical parameters are provided in the Supplementary Materials (Tables S8–S10).

#### 2.3.6. Effect of Temperature on Correlation Relationships

Extraction temperature exhibited a non-uniform influence on the relationships between global antioxidant parameters and the concentrations of individual prenylated flavonoids. Correlation analysis revealed weak but statistically significant positive associations between extraction temperature and total phenolic content (TPC; Pearson *r* = 0.239, *p* < 0.001), as well as between temperature and total antioxidant capacity (TAC; *r* = 0.153, *p* < 0.001), indicating a general tendency toward higher global antioxidant indicators at elevated extraction temperatures.

In contrast, compound-specific behavior was observed for individual prenylated flavonoids. A weak to moderate positive correlation was identified between extraction temperature and isoxanthohumol (IXN) concentration (r = 0.203, *p* < 0.001). The relationship between temperature and 8-prenylnaringenin (8-PN) was weaker but remained statistically significant (r = 0.096, *p* = 0.012).

No significant linear correlation was detected between extraction temperature and xanthohumol (XN) concentration using Pearson’s correlation coefficient (*r* = −0.047, *p* = 0.229). However, non-parametric analysis revealed a weak negative monotonic association between temperature and XN concentration (Spearman’s ρ = −0.125, *p* = 0.001), indicating a non-linear temperature-dependent response.

Overall, these results demonstrate that extraction temperature influences global antioxidant indicators and individual prenylated flavonoids in a compound-dependent manner. While temperature-related increases in TPC and TAC were detectable at the extract level, the direction and strength of temperature–compound relationships differed among individual prenylated flavonoids.

### 2.4. Multivariate Analysis (Principal Component Analysis, PCA)

**Figure 6:**
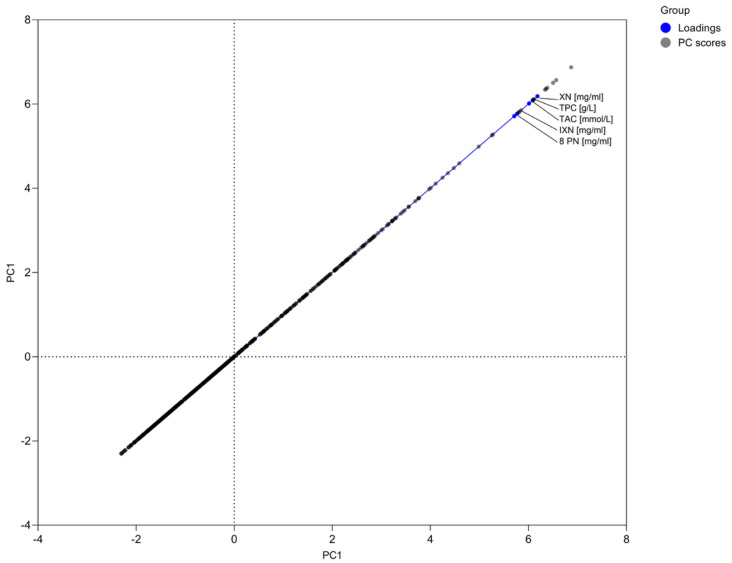
Principal component analysis (PCA) of global antioxidant parameters and prenylated flavonoids in hop extracts. The score plot illustrates the distribution of samples according to extraction conditions, while the separation of variables reflects shared and compound-specific chemical trends. Global antioxidant parameters cluster together, whereas extraction temperature emerges as an independent technological driver influencing extract composition.

Principal component analysis (PCA) was applied to explore multivariate relationships among global antioxidant indicators (TPC and TAC), concentrations of prenylated flavonoids, and extraction temperature across all hop varieties and extraction conditions (Figure 6). PCA enabled visualization of shared variability and dominant trends within the dataset that could not be resolved by univariate analysis alone.

The first principal component (PC1) explained the largest proportion of total variance in the dataset and was primarily associated with extraction temperature and global antioxidant indicators. Samples extracted at higher temperatures were clearly separated along PC1, reflecting the strong influence of temperature on TPC and TAC values. This separation was consistent across hop varieties and extraction solvents, confirming extraction temperature as the dominant multivariate driver of extract composition.

The second principal component (PC2) accounted for a smaller but distinct proportion of variance and was mainly associated with differences in prenylated flavonoid profiles. In particular, opposing loadings of xanthohumol and isoxanthohumol contributed to sample differentiation along PC2, reflecting compound-specific responses to extraction temperature and processing conditions. Samples with higher xanthohumol content clustered separately from those enriched in isoxanthohumol, consistent with the trends observed in targeted HPLC-DAD analysis.

Cryogenic homogenization and solvent type contributed to secondary clustering patterns within the PCA space but did not override the dominant temperature-driven separation. Inclusion of extraction temperature as an explanatory variable confirmed its central role in shaping both the global antioxidant response and compound-specific behavior.

Overall, PCA supported the conclusions derived from univariate and correlation analyses by demonstrating that global antioxidant indicators and individual prenylated flavonoids respond to extraction parameters in a partially overlapping but non-identical manner. Complete PCA score and loading matrices, including the proportion of variance explained by each principal component, are provided in the Supplementary Materials.

## 3. Discussion

This section discusses the experimental findings in the context of extraction chemistry, compound stability, and methodological considerations. The results obtained from global antioxidant assays are interpreted alongside targeted chromatographic and multivariate analyses to elucidate the relationships between technological parameters and the molecular composition of hop extracts.

### 3.1. Interpretation of Global Antioxidant Indicators Under ASE Conditions

The present study demonstrates that global antioxidant indicators, namely total phenolic content (TPC) and total antioxidant capacity (TAC), respond systematically to changes in accelerated solvent extraction (ASE) parameters. Across all hop varieties and extraction conditions, extraction temperature emerged as the dominant technological factor influencing both parameters, while homogenization method and solvent type exerted secondary but statistically significant effects. Similar temperature-driven responses of global antioxidant indicators have been reported for hop-derived extracts and other plant matrices processed under high-pressure or elevated-temperature extraction conditions [5,7,8,9,10,11].

The strong correlation observed between TPC and TAC across the dataset supports their coherent analytical behavior and reflects their shared sensitivity to the cumulative pool of antioxidant-active constituents in hop extracts. This agreement is consistent with previous reports demonstrating close associations between phenolic content and antioxidant capacity in hop extracts and related plant materials [2,10,12,13,14]. As both assays are inherently integrative, they capture the combined reducing and radical-scavenging capacity of chemically diverse extract components rather than the behavior of individual compounds [11,14].

However, the present results also indicate that increases in TPC and TAC with extraction temperature do not necessarily imply proportional preservation of specific bioactive constituents. At elevated temperatures, global antioxidant indicators continued to increase or stabilize, whereas targeted chromatographic analysis revealed compound-specific trends, including degradation or transformation of thermolabile prenylated flavonoids. Similar discrepancies between global antioxidant responses and compound-specific stability have been described for prenylated flavonoids and other phenolic subclasses subjected to thermal or high-pressure processing [1,2,6,15,16,17].

From a practical perspective, these findings suggest that TPC and TAC are well suited for rapid screening and comparative optimization of extraction efficiency but should be interpreted cautiously when used as proxies for compound-specific quality attributes. When extraction strategies aim to preserve or enrich defined bioactive molecules such as prenylated flavonoids, reliance on global antioxidant assays alone may therefore be insufficient, and targeted chromatographic analysis becomes essential to ensure meaningful process control and product standardization [1,3,10,18,19].

### 3.2. Temperature-Driven Transformation and Stability of Prenylated Flavonoids

The targeted HPLC-DAD analysis revealed pronounced, compound-specific responses of prenylated flavonoids to extraction temperature under accelerated solvent extraction conditions. Among the investigated compounds, xanthohumol (XN) and isoxanthohumol (IXN) exhibited opposing temperature-dependent trends, whereas 8-prenylnaringenin (8-PN) showed a narrower temperature optimum and lower overall stability. This compound-dependent behavior is consistent with broader literature describing distinct stability and reactivity patterns among hop prenylflavonoids during processing [10,18,19,20].

The decrease in xanthohumol concentration observed at elevated extraction temperatures was accompanied by a concomitant increase in isoxanthohumol. This behavior is consistent with temperature-induced isomerization of xanthohumol to isoxanthohumol, a process that has been well documented in hop-derived matrices and during thermal processing of hops and hop products [1,2,18,20,21]. Under high-temperature ASE conditions, this transformation appears to be promoted not only by increased molecular mobility but also by prolonged exposure of prenylated chalcones to elevated thermal energy, resulting in partial conversion to the corresponding flavanone. Related work on XN stability and degradation mechanisms further supports the sensitivity of XN to processing temperature and storage/processing conditions [15,16,17].

In contrast to XN and IXN, 8-prenylnaringenin was detected at substantially lower concentrations and exhibited a more limited temperature tolerance. Maximum 8-PN levels were observed at intermediate extraction temperatures, whereas further temperature increase resulted in a gradual decline. This behavior is consistent with previous reports describing the thermal instability of 8-PN and its susceptibility to degradation or secondary transformation under elevated temperatures [1,18,19,22]. Given its potent estrogenic activity, even small changes in 8-PN concentration may be biologically relevant, underscoring the importance of temperature control when targeting this compound.

These compound-specific temperature responses explain, at least in part, the moderate correlations observed between global antioxidant indicators and individual prenylated flavonoids. While increasing extraction temperature enhanced global antioxidant responses at the extract level, it simultaneously promoted chemical transformations that altered the relative composition of prenylated flavonoids. Similar decoupling of global antioxidant capacity from individual phenolic stability has been reported in thermally processed plant extracts and highlights the limitations of linear models when interpreting data across wide temperature ranges [6,11,23,24].

Taken together, these findings emphasize that extraction temperature represents a critical control parameter governing not only extraction efficiency but also chemical integrity of prenylated flavonoids. Optimization strategies based solely on global antioxidant indicators may therefore overlook temperature-driven transformations that substantially modify the bioactive profile of hop extracts.

### 3.3. Implications for Process Optimization and Method Selection: Global Versus Targeted Approaches

The combined evaluation of global antioxidant indicators and targeted chromatographic data provides important insights for the rational selection of analytical strategies in hop extraction and processing. While total phenolic content (TPC) and total antioxidant capacity (TAC) responded consistently to changes in extraction parameters, their integrative nature limits their ability to resolve compound-specific behavior, particularly under conditions that promote chemical transformation of thermolabile constituents [10,11,14].

The present results indicate that extraction temperatures in the range of approximately 100–150 °C represent a practical compromise between maximizing global antioxidant response and maintaining favorable prenylated flavonoid profiles. Within this temperature window, TAC and TPC reached near-maximum values, while xanthohumol concentrations remained relatively high and excessive conversion to isoxanthohumol was limited. Similar temperature-dependent trade-offs between extraction efficiency and compound stability have been reported for hop-derived phenolics and prenylated flavonoids subjected to thermal or high-pressure processing [5,6,9,10,25].

At higher extraction temperatures (≥200 °C), global antioxidant indicators remained elevated; however, targeted analysis revealed pronounced shifts in prenylated flavonoid composition, including depletion of xanthohumol and reduced stability of 8-prenylnaringenin. Under such conditions, reliance on TAC or TPC alone may therefore be misleading, as high global antioxidant values can coincide with unfavorable changes in the bioactive profile. This observation is consistent with previous studies demonstrating that global antioxidant assays may mask qualitative changes in phenolic composition when applied across wide processing ranges [2,6,11,16,23].

From an analytical and technological standpoint, these findings support a tiered decision framework. Global antioxidant assays such as TAC and TPC are well suited for rapid screening, comparative evaluation of extraction efficiency, and preliminary process optimization. In contrast, targeted chromatographic analysis becomes essential when process objectives include preservation of specific bioactive compounds, control of thermally induced transformations, or standardization of extract composition for functional or nutraceutical applications [1,3,10,18,19,22].

Importantly, the moderate correlations observed between global antioxidant indicators and individual prenylated flavonoids should not be interpreted as evidence of limited relevance of these compounds. Rather, they reflect the chemical heterogeneity of hop extracts and the cumulative nature of global assays, which integrate contributions from multiple phenolic subclasses and co-extracted constituents [10,11,14,19]. In this context, solvent–matrix interactions and solubility/dispersion phenomena in organic solvents can further modulate extraction selectivity, even for solvents of similar polarity, supporting the need for targeted profiling when optimizing solvent choice [26,27]. Finally, the present findings fit within broader trends toward intensified and greener extraction strategies, where method selection must balance yield, selectivity, and compound integrity [28,29].

## 4. Materials and Methods

### 4.1. Plant Material

Seven hop (*Humulus lupulus* L.) varieties harvested in 2023 from different cultivation regions were selected to cover a broad range of chemical composition and technological application (Table 1).

The varieties differed in their contents of α- and β-bitter acids as well as in their brewing use (aroma, bittering, or dual-purpose hops). To assess the influence of raw material processing, hop material obtained by cryogenic lupulin separation (CLS; variety Centennial) was also included. This technology employs liquid nitrogen to selectively separate lupulin glands enriched in bitter acids and aromatic compounds [30,31]. All hop samples were supplied by a certified producer (MAROMA s.r.o., Příbram, Czech Republic). The identity of each cultivar was verified based on supplier documentation and origin records. Prior to analysis, all samples were stored under dry and dark conditions at 4 °C to minimize chemical degradation.

### 4.2. Sample Preparation and Homogenization

Hop pellets were homogenized using two different approaches. Mechanical homogenization was performed by grinding 50 g of hop pellets in a porcelain mortar at ambient temperature. Cryogenic homogenization was carried out by immersing 50 g of pellets in 200 mL of liquid nitrogen, followed by grinding to a fine powder in a stainless-steel container. Cryogenically processed samples were either extracted immediately or stored at −80 °C until further analysis to prevent thermal or oxidative degradation.

### 4.3. Accelerated Solvent Extraction (ASE)

Accelerated solvent extraction was carried out using a Dionex ASE 350 system (Thermo Fisher Scientific, Waltham, MA, USA) operated at a constant pressure of 10.5 MPa to maintain solvents in the liquid state throughout the extraction process. Prior to extraction, 1 g of homogenized hop material was thoroughly mixed with 2 g of diatomaceous earth (Celite^®^, Sigma-Aldrich, St. Louis, MO, USA) to ensure uniform packing of the extraction cell. This procedure minimized channeling effects within the resin-rich lupulin matrix and enabled stable solvent flow under pressurized conditions.

Extractions were performed using ethanol or methanol (HPLC grade, 99.9% *v*/*v*; Merck, Darmstadt, Germany) as extraction solvents at temperatures of 50, 100, 150, and 200 °C. The selected temperature range was designed to encompass both moderate and elevated ASE conditions, allowing systematic evaluation of temperature-dependent extraction efficiency as well as potential thermally induced chemical transformations of prenylated flavonoids. Each extraction consisted of six consecutive static cycles of 5 min, corresponding to a total programmed extraction time of 30 min [9]. The use of multiple short static cycles was chosen to promote exhaustive extraction from the complex hop matrix while limiting prolonged thermal exposure of thermolabile compounds.

This extraction protocol is consistent with previously reported ASE applications for hop matrices and prenylated flavonoids, where multi-cycle extraction programs have been shown to improve compound recovery from resinous plant materials [6,9,18]. Following extraction, solvents were removed under reduced pressure, and the resulting dried extracts were stored at 4 °C until further analysis.

### 4.4. Determination of Total Phenolic Content—TPC

Total phenolic content (TPC) was determined using the Folin–Ciocalteu colorimetric method according to a previously described procedure with minor modifications [32]. Briefly, appropriately diluted extract samples were mixed with Folin–Ciocalteu reagent (Sigma-Aldrich, St. Louis, MO, USA), followed by neutralization with sodium carbonate. After incubation under controlled conditions, absorbance was measured spectrophotometrically at 765 nm (Shimadzu UV-1800, Kyoto, Japan).

Results were expressed as gallic acid equivalents (GAE) per unit mass of dry extract, based on an external calibration curve constructed using gallic acid standards (Sigma-Aldrich, St. Louis, MO, USA). Although the Folin–Ciocalteu assay is not selective exclusively for phenolic compounds and may respond to other reducing constituents, it is widely accepted as a robust indicator of the overall reducing and phenolic character of complex plant extracts.

### 4.5. Determination of Total Antioxidant Capacity—TAC

Total antioxidant capacity (TAC) was determined using the ABTS radical cation decolorization assay following established protocols [33]. The ABTS•^+^ radical was generated by the reaction of ABTS with potassium persulfate and allowed to stabilize prior to analysis. Diluted extract samples were reacted with the ABTS•^+^ solution, and the decrease in absorbance was recorded spectrophotometrically at 734 nm.

Antioxidant capacity was expressed as Trolox equivalents (TE), calculated from an external calibration curve prepared using Trolox standards (Sigma-Aldrich, St. Louis, MO, USA). The ABTS assay was selected due to its suitability for complex matrices containing both hydrophilic and lipophilic antioxidants, as well as its broad linear response range. In contrast to IC_50_-based metrics, TE-based quantification provides a more appropriate comparative measure for multicomponent plant extracts, where concentration-dependent inhibition curves may be difficult to interpret.

### 4.6. Quantitative Analysis of Prenylated Flavonoids by HPLC-DAD

Quantitative analysis of prenylated flavonoids—xanthohumol (XN), isoxanthohumol (IXN), and 8-prenylnaringenin (8-PN)—was performed using high-performance liquid chromatography coupled with diode-array detection (HPLC-DAD) on a Dionex Ultimate 3000 system (Thermo Fisher Scientific, Waltham, MA, USA). Chromatographic separation was achieved on a Kromasil C18 reversed-phase column (250 × 4.6 mm, 5 µm particle size, Supelco, Bellefonte, PA, USA) maintained at 25 °C.

For the determination of XN and IXN, an isocratic mobile phase consisting of water and methanol was employed. Analysis of 8-PN was carried out using an isocratic ternary mobile phase composed of water, methanol, and isopropanol, selected to ensure adequate resolution and peak symmetry for this compound. The flow rate was set to 1.0 mL·min^−1^, with total run times of 60 min for XN and IXN and 30 min for 8-PN. The injection volume was 20 µL.

Detection was performed at compound-specific wavelengths, namely 370 nm for XN, 290 nm for IXN, and 295 nm for 8-PN, based on their characteristic UV–Vis absorption maxima. Quantification was achieved using external calibration curves constructed from analytical reference standards over a concentration range of 0.0625–1.0 mg·mL^−1^, with coefficients of determination (R^2^) exceeding 0.995 for all analytes. Peak identity was confirmed by comparison of retention times and UV–Vis spectra with those of authentic standards [33].

Data acquisition and processing were conducted using Chromeleon™ 7.2 software (Thermo Fisher Scientific, Waltham, MA, USA). All measurements were performed in triplicate, and results are reported as mean values ± standard deviation.

### 4.7. Chemicals and Analytical Standards

All chemicals and reagents were of analytical grade. Xanthohumol, isoxanthohumol, and 8-prenylnaringenin standards (≥98% purity), as well as ethanol, methanol, and isopropanol (HPLC grade), were obtained from Sigma-Aldrich (Merck, Darmstadt, Germany). Deionized water was produced using a Milli-Q purification system (Merck Millipore, Billerica, MA, USA). Unless otherwise stated, all experiments and analyses were performed in triplicate.

### 4.8. Statistical Analysis

Statistical evaluation of the data was performed using Jamovi software (version 2.5; The Jamovi Project, Sydney, Australia). All experimental data were obtained from at least three independent measurements and are reported as mean values ± standard deviation. The effects of hop variety, extraction solvent, homogenization method, and extraction temperature on global antioxidant parameters (TPC and TAC) and individual prenylated flavonoid concentrations were assessed using analysis of variance (ANOVA). Where appropriate, multifactor models were applied to account for the combined influence of multiple extraction parameters.

Statistical significance was defined at *p* < 0.05. In addition to significance testing, effect sizes were evaluated using eta-squared (η^2^) values to assess the relative contribution of individual factors and their interactions to the observed variability. These effect size metrics are reported alongside *p*-values in the corresponding tables.

To investigate relationships between global antioxidant assays and targeted chromatographic data, correlation analysis was performed. Pearson’s correlation coefficient (*r*) was used to evaluate linear associations, while Spearman’s rank correlation coefficient (ρ) was applied as a complementary non-parametric measure to verify trend consistency, particularly in cases where non-linear responses to extraction temperature were observed. The strength of correlations was interpreted according to commonly accepted thresholds.

Multivariate relationships among global antioxidant parameters, prenylated flavonoid concentrations, and extraction temperature were further explored using principal component analysis (PCA). Prior to PCA, data suitability was confirmed using Bartlett’s test of sphericity and the Kaiser–Meyer–Olkin (KMO) measure of sampling adequacy. PCA was performed on standardized variables without rotation to identify dominant patterns in the dataset and to distinguish shared variability from process-driven effects. The proportion of variance explained by individual principal components is reported to facilitate interpretation.

## 5. Conclusions

This study systematically evaluated how global antioxidant indicators and targeted chromatographic profiling respond to changes in accelerated solvent extraction parameters applied to hop matrices. By combining total phenolic content (TPC) and total antioxidant capacity (TAC) with HPLC-DAD quantification of key prenylated flavonoids, we demonstrated that extraction temperature represents the dominant technological factor shaping both the global antioxidant response and compound-specific behavior. While TPC and TAC exhibited strong internal coherence and proved to be reliable indicators for comparative assessment of extraction efficiency, their integrative nature limited their ability to reflect changes in individual thermolabile prenylated flavonoids. In particular, extraction conditions that maximized global antioxidant indicators did not always coincide with optimal preservation of xanthohumol or 8-prenylnaringenin, highlighting a partial decoupling between the global antioxidant response and compound-specific stability at elevated temperatures. From a practical perspective, the results indicate that extraction temperatures in the range of approximately 100–150 °C provide a favorable balance between global antioxidant performance and maintenance of prenylated flavonoid profiles under ASE conditions. Global antioxidant assays are therefore well suited for rapid screening and preliminary process optimization, whereas targeted chromatographic analysis becomes essential when process objectives include control of temperature-driven transformations or standardization of specific bioactive constituents. Overall, this work emphasizes the complementary roles of global and targeted analytical approaches and provides a rational framework for selecting appropriate evaluation strategies in the optimization of hop extraction processes.

## Figures and Tables

**Table 1:** Characteristics of the hop varieties used.

Variety	Country of Origin	α-Bitter Acids (%)	β-Bitter Acids (%)	Hopping Type
Saaz Late	Czech Republic	2.69	4.0	Second and third hopping
Premiant	Czech Republic	7.3	3.5	Universal
Centennial Cryo	USA	11.7	4.5	Second and third hopping, dry hopping
Galaxy	Australia	13.6	5.2	Third and cold hopping
Styrian Wolf	Slovenia	14.9	6.0	Second and third hopping, dry hopping
Moutere	New Zealand	15.3	7.7	Universal
Polaris	Germany	17.6	6.0	Universal

## Data Availability

All data supporting the findings of this study are included in the manuscript and its Supplementary Materials.

## Supplementary Materials

The following supporting information can be downloaded at: https://www.mdpi.com/article/10.3390/molecules31030562/s1, The Supplementary Materials provide complete experimental datasets and extended statistical analyses supporting the results presented in the main manuscript. These include full datasets for total antioxidant capacity (TAC) and total polyphenol content (TPC) across all accelerated solvent extraction conditions and hop varieties (Tables S1–S4), as well as quantitative HPLC-DAD data for xanthohumol, isoxanthohumol, and 8-prenylnaringenin (Tables S5–S7). Extended correlation analyses and statistical outputs supporting the correlation and multivariate analyses are also provided.

## Abbreviations

The following abbreviations are used in this manuscript:

ASE	Accelerated Solvent Extraction
XN	Xanthohumol
IXN	Isoxanthohumol
8-PN	8-Prenylnaringenin
HPLC-DAD	High-Performance Liquid Chromatography with Diode-Array Detection
TAC	Total Antioxidant Capacity
ROS	Reactive Oxygen Species
ANOVA	Analysis of Variance
CLS	Cryogenic Lupulin Separation
KMO	Kaiser-Meyer-Olkin measure
PCA	Principal Component Analysis
PC	Principal Component
TPC	Total Polyphenol Content
