# Supplementary material for: Linking Global Antioxidant Assays with Targeted HPLC Profiling of Prenylated Flavonoids in Humulus lupulus L. Extracts Obtained by Accelerated Solvent Extraction
Source: Molecules. 2026 Feb 5;31(3):562. doi: 10.3390/molecules31030562 (PMC12899039; doi:10.3390/molecules31030562)
Supplement: Supplementary file 1 [file molecules-31-00562-s001.zip › molecules-4113502-supplementary.pdf]

Supplementary Materials

The Supplementary Materials provide complete experimental datasets, extended statistical analyses, and detailed statistical descriptors supporting the results presented in the main manuscript. These materials are intended to ensure data transparency and to enable a detailed evaluation of the effects of extraction parameters on both global antioxidant indicators and targeted prenylated flavonoid profiles in hop extracts.

Specifically, Tables S1–S4 summarize comprehensive datasets for total antioxidant capacity (TAC) and total polyphenol content (TPC) obtained under all evaluated accelerated solvent extraction conditions. Tables S5–S7 provide full quantitative HPLC-DAD datasets for individual prenylated flavonoids, including xanthohumol, isoxanthohumol, and 8-prenylnaringenin. Together, these data complement the representative results presented in the main manuscript and support the correlation and multivariate analyses discussed therein.

Table S1. Effect of extraction temperature and homogenization method on total antioxidant capacity (TAC) of ethanolic hop extracts.
Total antioxidant capacity (TAC) expressed as mmol·dm^{-3} Trolox equivalents (TE) in hop extracts obtained by accelerated solvent extraction (ASE) using ethanol as the extraction solvent. Samples were prepared from seven hop varieties subjected to mechanical or cryogenic homogenization and extracted at 50, 100, 150, and 200 °C. Data are reported as mean values ± standard deviation (SD) (n = 6). Low standard deviation values reflect high analytical repeatability of the ABTS assay under controlled ASE conditions.

High-pressure extraction of 1 g of dry hop matter using ethanol (relative permittivity 24)						
Hop variety and homogenisation method			50 °C	100 °C	150 °C	200 °C
			TAC ABTS. mmol.dm^{-3} TE	TAC ABTS. mmol.dm^{-3} TE	TAC ABTS. mmol.dm^{-3} TE	TAC ABTS. mmol.dm^{-3} TE
Saaz Late	Mechanical homogenisation	ẋ (n=6)	7.170	9.700	13.550	11.110
		σ	1.012	0.039	0.136	2.093
	Liquid nitrogen homogenisation	ẋ (n=6)	8.770	12.810	16.740	12.530
		σ	0.020	0.015	0.804	0.949
Premiant	Mechanical homogenisation	ẋ (n=6)	12.260	17.490	15.630	10.570
		σ	0.722	0.718	0.109	1.014
	Liquid nitrogen homogenisation	ẋ (n=6)	12.620	19.050	16.530	10.780
		σ	0.782	0.787	0.103	1.049
Cennential Cryo	Mechanical homogenisation	ẋ (n=6)	5.460	14.090	12.730	8.290
		σ	0.027	0.041	0.039	0.043
	Liquid nitrogen homogenisation	ẋ (n=6)	8.460	18.210	13.920	8.540
		σ	0.041	0.250	0.499	0.039
Galaxy	Mechanical homogenisation	ẋ (n=6)	11.270	14.150	16.260	11.580
		σ	0.176	0.033	2.177	0.222
	Liquid nitrogen homogenisation	ẋ (n=6)	12.450	17.530	16.610	11.760
		σ	0.776	0.103	1.274	0.219
Styrian Wolf		ẋ (n=6)	13.880	15.510	18.490	14.130

	Mechanical homogenisation	σ	0.721	0.837	1.373	0.560
	Liquid nitrogen homogenisation	ẋ (n=6)	14.140	18.530	22.230	18.190
		σ	0.587	0.651	0.650	0.659
Moutere	Mechanical homogenisation	ẋ (n=6)	12.990	16.640	20.160	13.790
		σ	0.164	1.345	0.560	0.188
	Liquid nitrogen homogenisation	ẋ (n=6)	14.410	20.150	23.190	16.750
		σ	0.527	0.624	1.116	0.523
Polaris	Mechanical homogenisation	ẋ (n=6)	15.650	22.350	25.530	13.310
		σ	0.809	0.397	0.885	0.702
	Liquid nitrogen homogenisation	ẋ (n=6)	15.960	25.980	28.400	20.960
		σ	0.681	1.132	0.611	0.574

Table S2. Effect of extraction temperature and homogenization method on total antioxidant capacity (TAC) of methanolic hop extracts.
Total antioxidant capacity (TAC) expressed as mmol·dm^{-3} Trolox equivalents (TE) in hop extracts obtained by accelerated solvent extraction (ASE) using methanol. Samples were prepared from seven hop varieties using mechanical or cryogenic homogenization and extracted at temperatures ranging from 50 to 200 °C. Results are presented as mean ± SD (n = 6). Low standard deviation values reflect high analytical repeatability of the ABTS assay under controlled ASE conditions.

High-pressure extraction of 1 g of dry hop matter using methanol (relative permittivity 33)						
Hop variety and homogenisation method			50 °C	100 °C	150 °C	200 °C
			TAC ABTS. mmol.dm^{-3} TE	TAC ABTS. mmol.dm^{-3} TE	TAC ABTS. mmol.dm^{-3} TE	TAC ABTS. mmol.dm^{-3} TE
Saaz Late	Mechanical homogenisation	ẋ (n=6)	11.750	14.400	10.300	11.230
		σ	0.444	0.625	0.745	1.319
	Liquid nitrogen homogenisation	ẋ (n=6)	12.210	18.180	10.900	16.400
		σ	0.916	0.577	0.705	1.861
Premiant	Mechanical homogenisation	ẋ (n=6)	11.730	19.380	15.660	10.720
		σ	0.425	0.425	0.889	1.255
	Liquid nitrogen homogenisation	ẋ (n=6)	12.090	19.800	17.230	13.090
		σ	0.666	1.207	1.705	0.518
Cennential Cryo	Mechanical homogenisation	ẋ (n=6)	5.670	13.740	7.400	5.890
		σ	0.032	0.041	0.070	0.054
	Liquid nitrogen homogenisation	ẋ (n=6)	8.550	17.020	19.950	12.330
		σ	0.101	0.621	0.670	0.108
Galaxy	Mechanical homogenisation	ẋ (n=6)	8.870	12.490	20.350	12.340
		σ	0.095	0.085	0.085	0.267
		ẋ (n=6)	10.700	13.330	21.270	13.570

	Liquid nitrogen homogenisation	σ	0.048	0.505	0.048	0.113
Styrian Wolf	Mechanical homogenisation	ẋ (n=6)	11.610	12.700	18.720	8.980
		σ	0.056	0.085	0.145	0.052
	Liquid nitrogen homogenisation	ẋ (n=6)	13.240	16.550	20.820	14.530
		σ	0.020	0.095	0.089	0.085
Moutere	Mechanical homogenisation	ẋ (n=6)	8.960	16.640	18.420	11.640
		σ	0.108	0.141	0.210	0.254
	Liquid nitrogen homogenisation	ẋ (n=6)	11.420	21.520	24.690	11.660
		σ	0.057	0.054	0.742	0.133
Polaris	Mechanical homogenisation	ẋ (n=6)	12.080	19.320	21.860	19.100
		σ	0.636	1.239	1.331	0.920
	Liquid nitrogen homogenisation	ẋ (n=6)	13.220	25.230	25.470	23.320
		σ	0.597	1.881	1.340	1.124

Table S3. Total polyphenol content (TPC) in ethanolic hop extracts obtained under different extraction conditions. Total polyphenol content (TPC). expressed as $g \cdot dm^{-3}$ gallic acid equivalents (GAE). determined in hop extracts obtained by accelerated solvent extraction (ASE) using ethanol. The effects of extraction temperature (50–200 °C) and homogenization method (mechanical vs. cryogenic) were evaluated across seven hop varieties. Values represent mean ± SD (n = 6). Low standard deviation values reflect high analytical repeatability of the Folin-Ciocalteu assay under controlled ASE conditions.

High-pressure extraction of 1 g of dry hop matter using ethanol (relative permittivity 24)						
Hop variety and homogenisation method			50 °C	100 °C	150 °C	200 °C
			Total polyphenol content. $g.dm^{-3}$ GAE	Total polyphenol content. $g.dm^{-3}$ GAE	Total polyphenol content. $g.dm^{-3}$ GAE	Total polyphenol content. $g.dm^{-3}$ GAE
Saaz Late	Mechanical homogenisation	ẋ (n=6)	0.730	1.200	1.110	0.980
		σ	0.056	0.030	0.033	0.071
	Liquid nitrogen homogenisation	ẋ (n=6)	1.020	1.430	1.410	1.040
		σ	0.044	0.059	0.053	0.079
Premiant	Mechanical homogenisation	ẋ (n=6)	0.810	1.120	1.280	1.000
		σ	0.031	0.048	0.029	0.180
	Liquid nitrogen homogenisation	ẋ (n=6)	1.200	1.500	1.580	1.110
		σ	0.028	0.042	0.097	0.112
Cennential (CSL)	Mechanical homogenisation	ẋ (n=6)	0.740	1.110	0.950	0.720
		σ	0.001	0.002	0.001	0.001
		ẋ (n=6)	1.550	1.720	1.650	1.020

	Liquid nitrogen homogenisation	σ	0.008	0.011	0.001	0.003
Galaxy	Mechanical homogenisation	ẋ (n=6)	1.020	1.630	1.480	1.250
		σ	0.002	0.004	0.004	0.003
	Liquid nitrogen homogenisation	ẋ (n=6)	1.160	1.930	2.030	1.330
		σ	0.003	0.045	0.005	0.006
Styrian Wolf	Mechanical homogenisation	ẋ (n=6)	1.000	1.890	2.200	1.610
		σ	0.001	0.001	0.002	0.009
	Liquid nitrogen homogenisation	ẋ (n=6)	1.320	2.110	2.520	2.190
		σ	0.004	0.002	0.003	0.003
Moutere	Mechanical homogenisation	ẋ (n=6)	0.940	2.380	2.080	1.170
		σ	0.001	0.006	0.002	0.003
	Liquid nitrogen homogenisation	ẋ (n=6)	1.120	2.450	2.570	1.980
		σ	0.002	0.001	0.003	0.011
Polaris	Mechanical homogenisation	ẋ (n=6)	1.180	2.330	2.510	2.050
		σ	0.021	0.037	0.030	0.068
	Liquid nitrogen homogenisation	ẋ (n=6)	1.180	2.650	2.570	2.070
		σ	0.025	0.040	0.059	0.034

Table S4. Total polyphenol content (TPC) in methanolic hop extracts obtained under different extraction conditions.
Total polyphenol content (TPC). expressed as $g \cdot dm^{-3}$ gallic acid equivalents (GAE). measured in hop extracts obtained by accelerated solvent extraction (ASE) using methanol as the extraction solvent. Samples were prepared from seven hop varieties subjected to mechanical or cryogenic homogenization and extracted at four temperatures (50–200 °C). Data are reported as mean ± SD (n = 6). Low standard deviation values reflect high analytical repeatability of the Folin-Ciocalteu assay under controlled ASE conditions.

High-pressure extraction of 1 g of dry hop matter using methanol (relative permittivity 33)						
Hop variety and homogenisation method			50 °C	100 °C	150 °C	200 °C
			Total polyphenol content. $g.dm^{-3}$ GAE	Total polyphenol content. $g.dm^{-3}$ GAE	Total polyphenol content. $g.dm^{-3}$ GAE	Total polyphenol content. $g.dm^{-3}$ GAE
Saaz Late	Mechanical homogenisation	ẋ (n=6)	0.630	1.100	0.750	0.740
		σ	0.024	0.049	0.023	0.030
	Liquid nitrogen homogenisation	ẋ (n=6)	1.130	1.380	1.150	0.740
		σ	0.037	0.055	0.052	0.048
Premiant	Mechanical homogenisation	ẋ (n=6)	0.570	1.180	1.290	1.290
		σ	0.019	0.043	0.044	0.096
		ẋ (n=6)	1.270	1.790	1.770	1.290

	Liquid nitrogen homogenisation	σ	0.027	0.035	0.093	0.110
Cennential Cryo	Mechanical homogenisation	ẋ (n=6)	0.570	1.620	1.090	0.600
		σ	0.001	0.002	0.004	0.002
	Liquid nitrogen homogenisation	ẋ (n=6)	1.270	1.930	1.110	1.900
		σ	0.000	0.000	0.000	0.000
Galaxy	Mechanical homogenisation	ẋ (n=6)	0.620	1.830	2.030	1.140
		σ	0.001	0.002	0.002	0.003
	Liquid nitrogen homogenisation	ẋ (n=6)	0.880	1.890	2.340	1.320
		σ	0.001	0.003	0.003	0.002
Styrian Wolf	Mechanical homogenisation	ẋ (n=6)	0.770	1.920	2.270	1.750
		σ	0.004	0.002	0.414	0.011
	Liquid nitrogen homogenisation	ẋ (n=6)	0.840	1.950	2.430	1.840
		σ	0.003	0.003	0.077	0.004
Moutere	Mechanical homogenisation	ẋ (n=6)	0.730	1.840	2.360	1.060
		σ	0.001	0.007	0.003	0.007
	Liquid nitrogen homogenisation	ẋ (n=6)	0.990	1.890	2.490	1.440
		σ	0.003	0.004	0.004	0.008
Polaris	Mechanical homogenisation	ẋ (n=6)	1.080	2.280	2.510	2.000
		σ	0.030	0.043	0.052	0.127
	Liquid nitrogen homogenisation	ẋ (n=6)	1.170	2.330	2.570	2.000
		σ	0.016	0.079	0.055	0.057

Table S5. Full dataset of xanthohumol (XN) concentrations in hop extracts obtained by accelerated solvent extraction.

Concentrations of xanthohumol (XN) ($mg \cdot mL^{-1}$) in hop extracts prepared by accelerated solvent extraction (ASE) under different combinations of solvent (ethanol or methanol). homogenization method (mechanical or cryogenic). extraction temperature (50–200 °C). and hop variety. Results are expressed as mean ± SD (n = 3). HPLC-DAD analyses were performed in triplicate due to instrumental and sample throughput considerations. The table also summarizes effect sizes (η^2) derived from multifactor ANOVA. indicating the relative contribution of technological factors to XN variability.

Variety	Homogenization	Solvent	50 °C	100 °C	150 °C	200 °C
Saaz Late	Mechanical	Ethanol	0.000 ± 0.000	0.023 ± 0.008	0.000 ± 0.000	0.042 ± 0.005
Saaz Late	Cryogenic	Ethanol	0.084 ± 0.000	0.051 ± 0.008	0.018 ± 0.004	0.000 ± 0.000
Premiant	Mechanical	Ethanol	0.042 ± 0.000	0.039 ± 0.008	0.010 ± 0.001	0.021 ± 0.002
Premiant	Cryogenic	Ethanol	0.002 ± 0.000	0.016 ± 0.003	0.022 ± 0.003	0.018 ± 0.002

Centennial (CSL)	Mechanical	Ethanol	0.044 ± 0.004	0.057 ± 0.012	0.033 ± 0.004	0.006 ± 0.002
Centennial (CSL)	Cryogenic	Ethanol	0.451 ± 0.013	1.180 ± 0.066	1.010 ± 0.047	0.920 ± 0.055
Galaxy	Mechanical	Ethanol	2.241 ± 0.100	1.582 ± 0.129	2.881 ± 0.115	2.285 ± 0.117
Galaxy	Cryogenic	Ethanol	2.444 ± 0.139	1.422 ± 0.097	3.354 ± 0.128	1.920 ± 0.092
Styrian Wolf	Mechanical	Ethanol	1.359 ± 0.182	1.872 ± 0.109	2.373 ± 0.111	0.026 ± 0.003
Styrian Wolf	Cryogenic	Ethanol	1.025 ± 0.131	1.392 ± 0.104	2.078 ± 0.102	0.021 ± 0.003
Moutere	Mechanical	Ethanol	2.322 ± 0.116	2.188 ± 0.115	2.782 ± 0.116	2.133 ± 0.117
Moutere	Cryogenic	Ethanol	2.857 ± 0.140	2.706 ± 0.136	3.046 ± 0.138	1.935 ± 0.094
Polaris	Mechanical	Ethanol	2.532 ± 0.111	2.475 ± 0.111	2.963 ± 0.109	1.600 ± 0.122
Polaris	Cryogenic	Ethanol	5.765 ± 0.158	5.547 ± 0.123	6.998 ± 0.125	1.877 ± 0.109
Saaz Late	Mechanical	Methanol	0.000 ± 0.000	0.013 ± 0.004	0.000 ± 0.000	0.015 ± 0.002
Saaz Late	Cryogenic	Methanol	0.049 ± 0.005	0.000 ± 0.000	0.035 ± 0.004	0.017 ± 0.002
Premiant	Mechanical	Methanol	0.012 ± 0.002	0.035 ± 0.004	0.019 ± 0.003	0.016 ± 0.002
Premiant	Cryogenic	Methanol	0.014 ± 0.002	0.000 ± 0.000	0.027 ± 0.003	0.012 ± 0.002
Centennial (CSL)	Mechanical	Methanol	0.012 ± 0.002	0.022 ± 0.003	0.014 ± 0.002	0.008 ± 0.001
Centennial (CSL)	Cryogenic	Methanol	0.068 ± 0.006	0.066 ± 0.005	0.047 ± 0.004	0.033 ± 0.003
Galaxy	Mechanical	Methanol	0.098 ± 0.008	0.084 ± 0.007	0.077 ± 0.007	0.053 ± 0.005
Galaxy	Cryogenic	Methanol	0.121 ± 0.010	0.091 ± 0.008	0.065 ± 0.006	0.044 ± 0.004
Styrian Wolf	Mechanical	Methanol	0.040 ± 0.004	0.033 ± 0.003	0.025 ± 0.003	0.018 ± 0.002
Styrian Wolf	Cryogenic	Methanol	0.029 ± 0.003	0.033 ± 0.003	0.026 ± 0.003	0.017 ± 0.002
Moutere	Mechanical	Methanol	0.023 ± 0.003	0.042 ± 0.004	0.040 ± 0.003	0.023 ± 0.003
Moutere	Cryogenic	Methanol	0.022 ± 0.002	0.000 ± 0.000	0.037 ± 0.003	0.023 ± 0.003
Polaris	Mechanical	Methanol	0.784 ± 0.051	0.935 ± 0.051	0.553 ± 0.053	0.239 ± 0.027
Polaris	Cryogenic	Methanol	2.007 ± 0.100	1.884 ± 0.107	0.739 ± 0.047	0.604 ± 0.051

Table S6. Full dataset of isoxanthohumol (IXN) concentrations in hop extracts obtained by accelerated solvent extraction.

Concentrations of isoxanthohumol (IXN) (mg·mL^{-1}) determined in hop extracts obtained by accelerated solvent extraction (ASE) using different solvents. homogenization methods. extraction temperatures. and hop varieties. Values are reported as mean ± SD (n = 3). HPLC-DAD analyses were performed in triplicate due to instrumental and sample throughput considerations. Effect sizes (η^2) from multifactor ANOVA are provided to illustrate the influence of individual technological parameters.

**Variety**	**Homogenization**	**Solvent**	**50 °C**	**100 °C**	**150 °C**	**200 °C**
Saaz Late	Mechanical	Ethanol	0.000 ± 0.000	0.000 ± 0.000	0.000 ± 0.000	0.339 ± 0.027
Saaz Late	Cryogenic	Ethanol	0.604 ± 0.051	0.133 ± 0.012	0.034 ± 0.003	0.231 ± 0.027
Premiant	Mechanical	Ethanol	0.001 ± 0.000	0.015 ± 0.002	0.004 ± 0.001	0.097 ± 0.009
Premiant	Cryogenic	Ethanol	0.004 ± 0.001	0.004 ± 0.001	0.005 ± 0.001	0.009 ± 0.002
Centennial (CSL)	Mechanical	Ethanol	0.343 ± 0.027	0.372 ± 0.027	1.024 ± 0.054	1.562 ± 0.105
Centennial (CSL)	Cryogenic	Ethanol	0.000 ± 0.000	0.000 ± 0.000	0.180 ± 0.020	0.290 ± 0.027
Galaxy	Mechanical	Ethanol	0.207 ± 0.023	0.212 ± 0.023	0.044 ± 0.004	0.294 ± 0.027
Galaxy	Cryogenic	Ethanol	0.029 ± 0.003	0.506 ± 0.027	0.540 ± 0.026	1.029 ± 0.055
Styrian Wolf	Mechanical	Ethanol	0.451 ± 0.028	0.321 ± 0.027	0.379 ± 0.028	0.707 ± 0.052
Styrian Wolf	Cryogenic	Ethanol	0.408 ± 0.029	0.245 ± 0.026	0.235 ± 0.026	0.594 ± 0.053
Moutere	Mechanical	Ethanol	0.313 ± 0.027	0.108 ± 0.015	0.031 ± 0.004	0.503 ± 0.035
Moutere	Cryogenic	Ethanol	0.274 ± 0.026	0.053 ± 0.010	0.037 ± 0.004	0.394 ± 0.030
Polaris	Mechanical	Ethanol	0.498 ± 0.029	0.441 ± 0.027	0.419 ± 0.027	0.793 ± 0.054
Polaris	Cryogenic	Ethanol	1.096 ± 0.059	1.731 ± 0.105	1.200 ± 0.077	1.852 ± 0.104
Saaz Late	Mechanical	Methanol	0.009 ± 0.002	0.013 ± 0.003	0.005 ± 0.001	0.007 ± 0.001
Saaz Late	Cryogenic	Methanol	0.296 ± 0.026	0.214 ± 0.024	0.286 ± 0.026	0.098 ± 0.012
Premiant	Mechanical	Methanol	0.000 ± 0.000	0.003 ± 0.001	0.000 ± 0.000	0.003 ± 0.001
Premiant	Cryogenic	Methanol	0.199 ± 0.024	0.168 ± 0.021	0.124 ± 0.015	0.091 ± 0.012
Centennial (CSL)	Mechanical	Methanol	0.048 ± 0.008	0.041 ± 0.008	0.323 ± 0.027	0.452 ± 0.028
Centennial (CSL)	Cryogenic	Methanol	0.029 ± 0.004	0.020 ± 0.003	0.288 ± 0.026	0.345 ± 0.026

Galaxy	Mechanical	Methanol	0.218 ± 0.024	0.197 ± 0.024	0.259 ± 0.027	0.146 ± 0.020
Galaxy	Cryogenic	Methanol	0.167 ± 0.021	0.184 ± 0.022	0.207 ± 0.023	0.079 ± 0.010
Styrian Wolf	Mechanical	Methanol	0.290 ± 0.027	0.333 ± 0.028	0.289 ± 0.027	0.007 ± 0.001
Styrian Wolf	Cryogenic	Methanol	0.194 ± 0.023	0.170 ± 0.020	0.224 ± 0.026	0.006 ± 0.001
Moutere	Mechanical	Methanol	0.001 ± 0.000	0.269 ± 0.027	0.004 ± 0.001	0.403 ± 0.028
Moutere	Cryogenic	Methanol	0.427 ± 0.029	0.140 ± 0.020	0.024 ± 0.003	0.269 ± 0.027
Polaris	Mechanical	Methanol	0.153 ± 0.020	0.102 ± 0.009	0.101 ± 0.009	0.079 ± 0.008
Polaris	Cryogenic	Methanol	0.834 ± 0.053	1.016 ± 0.052	0.791 ± 0.053	0.675 ± 0.051

Table S7. Full dataset of 8-prenylnaringenin (8-PN) concentrations in hop extracts obtained by accelerated solvent extraction.

Concentrations of 8-prenylnaringenin (8-PN) (mg·mL^{-1}) measured in hop extracts obtained by accelerated solvent extraction (ASE) under varying solvent polarity. homogenization method. extraction temperature. and hop variety. Data are presented as mean ± SD (n = 3). HPLC-DAD analyses were performed in triplicate due to instrumental and sample throughput considerations. Multifactor ANOVA effect sizes (η^2) indicate the relative impact of extraction parameters on 8-PN yield.

Variety	Homogenization	Solvent	50 °C	100 °C	150 °C	200 °C
Saaz Late	Mechanical	Ethanol	0.187 ± 0.020	0.000 ± 0.000	0.000 ± 0.000	0.077 ± 0.007
Saaz Late	Cryogenic	Ethanol	0.122 ± 0.012	0.000 ± 0.000	0.082 ± 0.008	0.095 ± 0.007
Premiant	Mechanical	Ethanol	0.000 ± 0.000	0.000 ± 0.000	0.215 ± 0.020	0.141 ± 0.012
Premiant	Cryogenic	Ethanol	0.312 ± 0.024	0.118 ± 0.011	0.156 ± 0.014	0.161 ± 0.012
Centennial (CSL)	Mechanical	Ethanol	0.087 ± 0.010	0.397 ± 0.028	0.108 ± 0.009	0.102 ± 0.009
Centennial (CSL)	Cryogenic	Ethanol	0.000 ± 0.000	0.000 ± 0.000	0.000 ± 0.000	0.000 ± 0.000
Galaxy	Mechanical	Ethanol	0.000 ± 0.000	0.077 ± 0.007	0.101 ± 0.009	0.000 ± 0.000
Galaxy	Cryogenic	Ethanol	0.080 ± 0.008	0.492 ± 0.028	0.196 ± 0.020	0.260 ± 0.021
Styrian Wolf	Mechanical	Ethanol	0.000 ± 0.000	0.423 ± 0.028	0.122 ± 0.011	0.265 ± 0.021
Styrian Wolf	Cryogenic	Ethanol	0.000 ± 0.000	0.455 ± 0.029	0.152 ± 0.013	0.235 ± 0.020
Moutere	Mechanical	Ethanol	0.000 ± 0.000	0.201 ± 0.020	0.140 ± 0.013	0.195 ± 0.016

Moutere	Cryogenic	Ethanol	0.000 ± 0.000	0.538 ± 0.030	0.230 ± 0.020	0.279 ± 0.021
Polaris	Mechanical	Ethanol	0.000 ± 0.000	0.134 ± 0.012	0.262 ± 0.021	0.450 ± 0.028
Polaris	Cryogenic	Ethanol	0.101 ± 0.009	0.590 ± 0.029	0.506 ± 0.028	0.363 ± 0.024
Saaz Late	Mechanical	Methanol	0.000 ± 0.000	0.000 ± 0.000	0.000 ± 0.000	0.000 ± 0.000
Saaz Late	Cryogenic	Methanol	0.000 ± 0.000	0.000 ± 0.000	0.000 ± 0.000	0.000 ± 0.000
Premiant	Mechanical	Methanol	0.000 ± 0.000	0.000 ± 0.000	0.000 ± 0.000	0.000 ± 0.000
Premiant	Cryogenic	Methanol	0.000 ± 0.000	0.000 ± 0.000	0.000 ± 0.000	0.000 ± 0.000
Centennial (CSL)	Mechanical	Methanol	0.000 ± 0.000	0.000 ± 0.000	0.000 ± 0.000	0.000 ± 0.000
Centennial (CSL)	Cryogenic	Methanol	0.000 ± 0.000	0.000 ± 0.000	0.000 ± 0.000	0.000 ± 0.000
Galaxy	Mechanical	Methanol	0.000 ± 0.000	0.000 ± 0.000	0.000 ± 0.000	0.000 ± 0.000
Galaxy	Cryogenic	Methanol	0.000 ± 0.000	0.000 ± 0.000	0.000 ± 0.000	0.000 ± 0.000
Styrian Wolf	Mechanical	Methanol	0.000 ± 0.000	0.000 ± 0.000	0.000 ± 0.000	0.000 ± 0.000
Styrian Wolf	Cryogenic	Methanol	0.124 ± 0.010	0.000 ± 0.000	0.170 ± 0.013	0.143 ± 0.011
Moutere	Mechanical	Methanol	0.000 ± 0.000	0.000 ± 0.000	0.000 ± 0.000	0.000 ± 0.000
Moutere	Cryogenic	Methanol	0.092 ± 0.008	0.087 ± 0.008	0.095 ± 0.008	0.000 ± 0.000
Polaris	Mechanical	Methanol	0.000 ± 0.000	0.233 ± 0.022	0.000 ± 0.000	0.086 ± 0.008
Polaris	Cryogenic	Methanol	0.000 ± 0.000	0.000 ± 0.000	0.240 ± 0.021	0.105 ± 0.009

Table S8. Detailed correlation matrix between global antioxidant parameters. prenylated flavonoids. and extraction temperature. Pearson correlation coefficients (r) and corresponding p-values describing relationships between total antioxidant capacity (TAC). total phenolic content (TPC). prenylated flavonoids (xanthohumol—XN. isoxanthohumol—IXN. 8-prenylnaringenin—8-PN). and extraction temperature in hop extracts obtained by accelerated solvent extraction. Pearson correlation coefficients (r) were calculated using all experimental data points ($n \approx 670$). Statistical significance was set at $p < 0.05$. Correlations involving 8-prenylnaringenin should be interpreted with caution due to its low absolute concentration and limited contribution to global antioxidant parameters.

Parameter pair	Pearson r	p-value	Interpretation
TAC – TPC	0.766	< 0.001	Strong positive correlation
TAC – XN	0.450	< 0.001	Moderate positive correlation
TAC – IXN	0.315	< 0.001	Moderate positive correlation
TAC – 8-PN	0.365	< 0.001	Moderate positive correlation
TPC – XN	0.405	< 0.001	Moderate positive correlation

TPC – IXN	0.350	< 0.001	Moderate positive correlation
TPC – 8-PN	0.413	< 0.001	Moderate positive correlation
Temperature – TPC	0.239	< 0.001	Weak positive correlation
Temperature – TAC	0.153	< 0.001	Weak positive correlation
Temperature – XN	−0.047	0.229	Not significant
Temperature – IXN	0.203	< 0.001	Weak positive correlation
TEMPERATURE – 8-PN	0.096	0.012	WEAK POSITIVE CORRELATION

Table S9. Effects of technological factors on global antioxidant parameters and prenylated flavonoids. Summary of one-way ANOVA and multifactor analysis of covariance (MANCOVA) evaluating the effects of hop variety. extraction solvent. homogenization method. and extraction temperature on total antioxidant capacity (TAC). total phenolic content (TPC). and concentrations of prenylated flavonoids (xanthohumol—XN. isoxanthohumol—IXN. and 8-prenylnaringenin—8-PN). Significance was assessed at $p < 0.05$. Detailed F-values and degrees of freedom are available upon request. Multifactor analysis of covariance (MANCOVA) was applied to account for the combined effects of technological parameters and extraction temperature.

Response variable	Factor	Statistical test	Effect	p-value
TAC	Hop variety	ANOVA	Significant	< 0.001
TAC	Solvent	ANOVA	Significant	< 0.001
TAC	Homogenization	ANOVA	Significant	< 0.01
TAC	Extraction temperature	MANCOVA	Significant	< 0.001
TPC	Hop variety	ANOVA	Significant	< 0.001
TPC	Solvent	ANOVA	Significant	< 0.001
TPC	Homogenization	ANOVA	Significant	< 0.05
TPC	Extraction temperature	MANCOVA	Significant	< 0.001
XN	Hop variety	ANOVA	Significant	< 0.001
XN	Solvent	ANOVA	Significant	< 0.001
XN	Homogenization	ANOVA	Significant	< 0.01
XN	Extraction temperature	MANCOVA	Significant	< 0.001
IXN	Hop variety	ANOVA	Significant	< 0.001
IXN	Solvent	ANOVA	Significant	< 0.001
IXN	Homogenization	ANOVA	Significant	< 0.05
IXN	Extraction temperature	MANCOVA	Significant	< 0.001
8-PN	Hop variety	ANOVA	Significant	< 0.001
8-PN	Solvent	ANOVA	Significant	< 0.001
8-PN	Homogenization	ANOVA	Significant	< 0.05
8-PN	Extraction temperature	MANCOVA	Significant	< 0.01

Table S10. Principal component analysis (PCA) loadings and explained variance. Loadings of variables on the first principal component (PC1) obtained by principal component analysis (PCA) and their corresponding uniqueness values. PCA was performed to explore multivariate relationships between global antioxidant parameters. prenylated flavonoids. and extraction temperature in hop extracts obtained by accelerated solvent extraction. Bartlett's test of sphericity confirmed the suitability of the dataset for PCA ($\chi^2 = 1513$. $p < 0.001$). The Kaiser–Meyer–Olkin (KMO) measure of sampling adequacy was 0.692. indicating acceptable sampling adequacy. PCA was performed on standardized variables without rotation. Extraction temperature was treated as a supplementary variable and is therefore not associated with a PC1 loading.

Variable	PC1 loading	Uniqueness
Total phenolic content (TPC)	0.786	0.382
Total antioxidant capacity (TAC)	0.768	0.410

Variable	PC1 loading	Uniqueness
Xanthohumol (XN)	0.767	0.411
Isoxanthohumol (IXN)	0.740	0.453
8-Prenylnaringenin (8-PN)	0.723	0.478
Extraction temperature	—	0.936

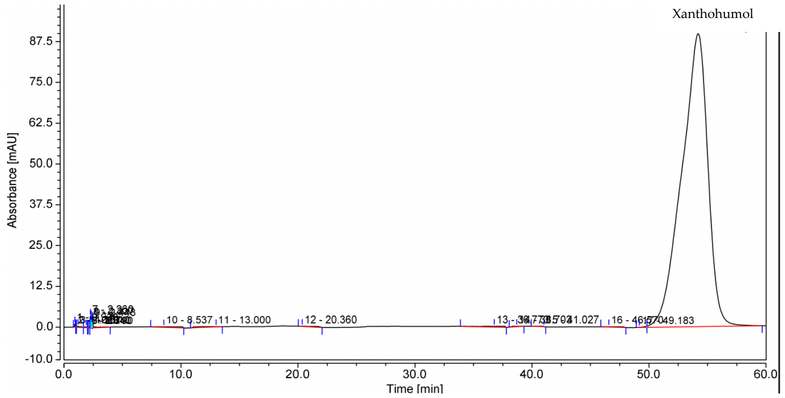

Figure S1. Representative HPLC-DAD chromatogram of the xanthohumol (XN) standard analyzed under identical chromatographic conditions as the hop extracts. The dominant peak corresponds to xanthohumol with a retention time of approximately RT ≈ 49.8 min. Detection was performed using a diode array detector at a wavelength of 370 nm. Compound identification was based on the comparison of retention time and UV–Vis spectral characteristics with the authentic standard.

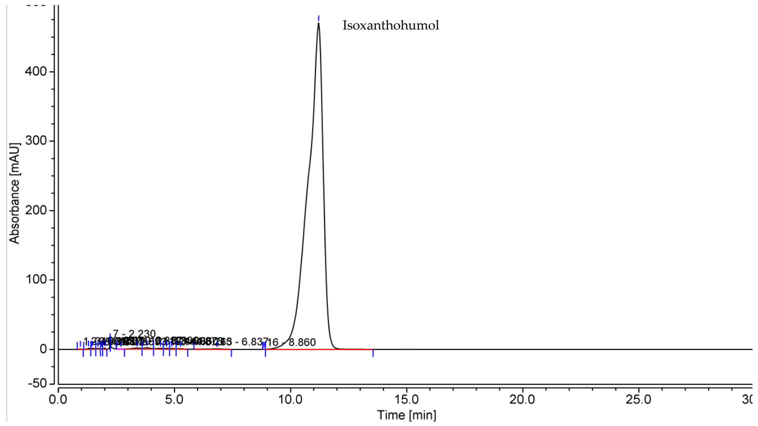

Figure S2. Representative HPLC-DAD chromatogram of the isoxanthohumol (IXN) standard analyzed under identical chromatographic conditions as the hop extracts. The dominant peak corresponds to isoxanthohumol with a retention time of approximately RT ≈ 11.2 min. Detection was performed using a diode array detector at a wavelength of 290 nm. Compound identification was based on the comparison of retention time and UV–Vis spectral characteristics with the authentic standard.

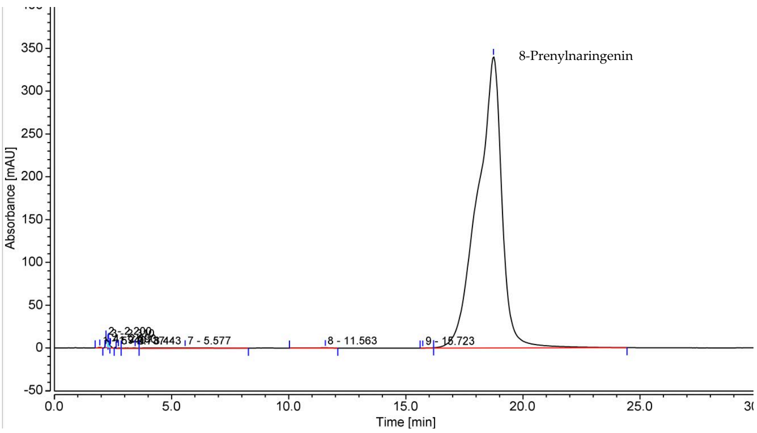

Figure S3. Representative HPLC-DAD chromatogram of the 8-prenylnaringenin (8-PN) standard analyzed under identical chromatographic conditions as the hop extracts. The dominant peak corresponds to 8-prenylnaringenin with a retention time of approximately RT ≈ 18.7 min. Detection was performed using a diode array detector at a wavelength of 295 nm. Compound identification was based on the comparison of retention time and UV–Vis spectral characteristics with the authentic standard.
